# Hg(II) Coordination Polymers Based on *N,N’*-bis(pyridine-4-yl)formamidine

**DOI:** 10.3390/polym8040137

**Published:** 2016-04-11

**Authors:** Wayne Hsu, Xiang-Kai Yang, Pradhumna Mahat Chhetri, Jhy-Der Chen

**Affiliations:** Department of Chemistry, Chung Yuan Christian University, Chung-Li 320, Taiwan, R. O. C.; wwffwayne@hotmail.com (W.H.); two882@yahoo.com.tw (X.-K.Y.); mahatp@gmail.com (P.M.C.)

**Keywords:** coordination polymer, mercury, formamidine

## Abstract

Reactions of *N,N’*-bis(pyridine-4-yl)formamidine (4-Hpyf) with HgX_2_ (X = Cl, Br, and I) afforded the formamidinate complex {[Hg(4-pyf)_2_]·(THF)}*_n_*, **1**, and the formamidine complexes {[HgX_2_(4-Hpyf)]·(MeCN)}*_n_* (X = Br, **2**; I, **3**), which have been structurally characterized by X-ray crystallography. Complex **1** is a 2D layer with the {4^4^·6^2^}-**sql** topology and complexes **2** and **3** are helical chains. While the helical chains of **2** are linked through N–H···Br hydrogen bonds, those of **3** are linked through self-complementary double N–H···N hydrogen bonds, resulting in 2D supramolecular structures. The 4-pyf- ligands of **1** coordinate to the Hg(II) ions through one pyridyl and one adjacent amine nitrogen atoms and the 4-Hpyf ligands of **2** and **3** coordinate to the Hg(II) ions through two pyridyl nitrogen atoms, resulting in new bidentate binding modes. Complexes **1**–**3** provide a unique opportunity to envisage the effect of the halide anions of the starting Hg(II) salts on folding and unfolding the Hg(II) coordination polymers. Density function theory (DFT) calculation indicates that the emission of **1** is due to intraligand π→π * charge transfer between two different 4-pyf- ligands, whereas those of **2** and **3** can be ascribed to the charge transfer from non-bonding p-type orbitals of the halide anions to π * orbitals of the 4-pyf- ligands (*n*→π *). The gas sorption properties of the desolvated product of **1** are compared with the Cu analogues to show that the nature of the counteranion and the solvent-accessible volume are important in determining their adsorption capability.

## 1. Introduction

Functional coordination polymers have been a research focus in recent years due to their potential applications in separation, ion exchange, catalysis, and adsorption [1,2,3,4,5]. Of these coordination polymers, the 1D and 2D ones that show the simple topological types of coordination arrays are found to be dominating the literature [6,7]. The relative simplicity of the 1D and 2D coordination polymers and their ease of formation through self-assembly facilitate the incorporation of functional properties at the metal centers and the backbone of the organic linkers. Although metal cations are essential common constituents of coordination polymers, the contribution of organic ligands in the design and construction of desired networks is highly appreciable due to their possible changes in flexibility, length, and symmetry [8,9].

**Figure 1:**
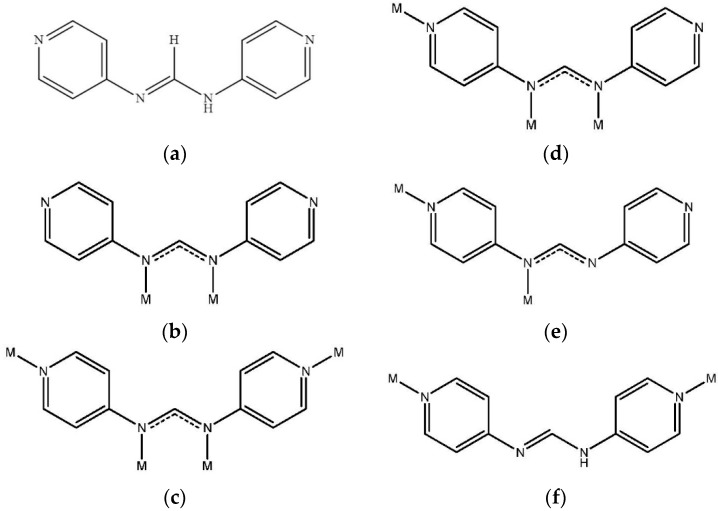
Binding modes of the anionic 4-pyf^−^ and neutral 4-Hpyf ligand. (**a**) Neutral ligand; (**b**) Bidentate bonding mode through two inner amine nitrogen atoms; (**c**) Tetradentate bonding mode; (**d**) Tridentate bonding mode; (**e**) Bidentate bonding mode through one amine and one pyridyl nitrogen atoms and (**f**) Bidentate bonding mode through two pyridyl nitrogen atoms.

The coordination chemistry of metal complexes containing neutral formamidine and anionic formamidinate ligands has been investigated extensively in recent years [10]. Mononuclear and polynuclear complexes have been reported [10,11,12,13]. These types of ligands could be improved by having different directions of donor sites to construct coordination polymers with higher dimensionality [14,15]. In this regard, we have designed and synthesized the dimolybdenum paddlewheel complex [Mo2(4-pyf)4][4-Hpyf = *N*,*N*’-bis(pyridine-4-yl)formamidine] (Figure 1a), which was reacted with Hg(II) salts to afford the first 2D and 3D heteronuclear coordination networks based on quadruple-bonded dimolybdenum units [14]. By one-pot solvothermal reactions of 4-aminopyridine and triethylorthoformate with divalent copper salts, several 2D coordination networks of the types *anti*-{[Cu(4-pyf)]·solvent}*_n_* and *syn*-{[Cu_4_(4-pyf)_4_]·2solvent}*_n_* (solvent = MeOH and EtOH) that show crystal-to-crystal transformations and photoluminescence changes have also been reported [15]. In these complexes, the anionic 4-pyf- ligands show bidentate binding mode through two inner amine nitrogen atoms, Figure 1b, tetradentate binding mode through all the four nitrogen atoms, Figure 1c, or tridentate binding mode with one dangling pyridyl nitrogen atom, Figure 1d.

As our continuing efforts to investigate the correlation between the binding modes of formamidinate ligand and the structural diversity of novel coordination polymers, we sought to investigate the halide anion effect of metal salts on the construction of formamidinate ligand-based Hg(II) coordination polymers. The synthesis, structures, and luminescence and adsorption properties of {[Hg(4-pyf)_2_]·(THF)}*_n_*, **1**, {[HgBr_2_(4-Hpyf)]·(ACN)}*_n_*, **2**, and {[HgI_2_(4-Hpyf)]·(ACN)}*_n_*, **3**, form the subject of this report. New types of bidentate binding modes, Figure 1e and Figure 1f, are found for the anionic and neutral 4-Hpyf ligands. The halide anions show significant effect on the structural diversity, and complexes **1**–**3** represent a unique example in folding and unfolding the Hg(II) coordination polymers. Moreover, the formation of complex **1**, in which the 4-pyf- ligand adopts the binding mode of Figure 1e, provides an opportunity to investigate the effect of the dangling pyridyl nitrogen atoms on the pore structure and gas storage capability.

## 2. Experimental Section

### 2.1. General Procedures

Elemental analyses were carried out using an Elementar Vario EL cube analyzer (Elementar Analysensysteme GmbH, Hanau, Germany). IR spectra (KBr disk) were recorded on a Jasco FT/IR-460 plus spectrometer (Jasco, Easton, PA, USA). Emission spectra were recorded on a Hitachi F-4500 spectrometer (Hitachi, Tokyo, Japan). Powder X-ray diffraction measurements were measured using a PANalytical PW3040/60 X’Pert Pro diffractometer (PANalytical, EA Almelo, Netherlands) or a Bruker D2 PHASER X-ray diffractometer (Bruker Corporation, Karlsruhe, Germany). ^1^H NMR spectra were recorded on a Bruker Avance II 400 MHz FT-NMR spectrometer (Bruker Corporation, Rheinstetten, Germany) by using DMSO-d_6_ as an internal standard.

### 2.2. Materials

The reagent 4-aminopyridine was purchased from Alfa Aesar (Lancashire, UK ), mercury (II) chloride and mercury(II) bromine from ACROS (Pittsburgh, PA, USA.), and mercury(II) iodine and triethyl orthoformate from Sigma-Aldrich Co. (St. Louis, MI, USA). The complexes *syn*-{[Cu_4_(4-pyf)_4_]·2EtOH}*_n_*, *syn*-{[Cu_3_(4-pyf)_2_](BF_4_)·2H_2_O·EtOH}*_n_*, and *syn*-{[Cu_3_(4-pyf)_2_](ClO_4_)·EtOH}*_n_* were prepared according to reported procedures [15].

### 2.3. Preparations

#### 2.3.1. Synthesis of 4-Hpyf

Triethyl orthoformate (12 mL, 0.07 mol) was added to a round-bottom flask containing 4-aminopyridine (9.50 g, 0.10 mol) and equipped with a condenser. The mixture was then stirred at 140 °C for 24 h to yield a brown solution and khaki precipitation. The precipitate was filtered, washed with 100 mL of diethylether, and dried under vacuum. Yield: 8.82 g (89%). ^1^H NMR (400 MHz, δ, ppm in DMSO-d_6_): 10.45 (s, 1 H, N–H), 8.48 (s, 1 H, C–H), 8.38 (d, 4 H, py), 7.23 (d, 4 H, py). Anal. Calcd. for C_11_H_10_N_4_ (*M*_W_ = 198.23): C, 66.65; H, 5.08; N, 28.26%. Found: C, 66.57; H, 4.99; N, 28.29%. IR (KBr disk, cm^-1^): 1718 (m), 1655 (s), 1605 (s), 1567 (s), 1491 (s), 1417 (m), 1379 (m), 1350 (m), 1311 (s), 1263 (m), 1222 (s), 1191 (s), 1119 (m), 1100 (w), 646 (w), 536 (m). GC-MS: *m*/*z* found 198. The ^1^H NMR spectrum is shown in Figure S1 (Supplementary Material). Crystals of 4-Hpyf·0.16THF suitable for X-ray structural analysis were obtained by slow diffusion of diethyl ether into a THF solution of 4-Hpyf.

#### 2.3.2. Synthesis of {[Hg(4-pyf)_2_]·2(THF)}_n_, **1**

4-Hpyf (0.40 g, 2.02 mmol) and HgCl_2_ (0.27 g, 0.99 mmol) were placed in a flask containing 20 mL of THF. The mixture was then stirred at room temperature for 24 h to produce a colorless solution and a colorless solid. The solution was filtered and diethylether was added to introduce a precipitate. The precipitate was filtered and washed with diethyl ether (2 × 10 mL) and THF (2 × 10 mL) and then dried under vacuum to produce a white product. Yield: 0.38 g (51%). Anal. Calcd. for C_30_H_34_HgN_8_O_2_ (*M*_W_ = 739.24): C, 48.74; H, 4.64; N, 15.16%. Found: C, 48.20; H, 4.53; N, 14.77%. IR (KBr disk, cm^-1^): 3365(m), 2359(w), 1654(m), 1574(s), 1541(s), 1507(m), 1423(w), 1312(m), 1248(w), 1006(w), 828(w), 669(w), 419(w). Crystals of **1** suitable for X-ray structural analysis were obtained by layering a MeOH solution of HgCl_2_ with a THF solution of 4-Hpyf.

#### 2.3.3. Synthesis of {[HgBr_2_(4-Hpyf)]·(CH_3_CN)}*_n_*, **2**, and {[HgI_2_(4-Hpyf)]·(CH_3_CN)}*_n_*, **3**

4-Hpyf (0.20 g, 1.01 mmol) was placed in a flask containing 20 mL of acetonitrile and then HgBr_2_ (0.36 g, 1.00 mmol) or HgI_2_ (0.45 g, 0.99 mmol) was added. The mixture was then stirred at room temperature for 24 h to produce a colorless solution and a colorless solid. The solution was filtered and diethylether was added to introduce precipitate. The precipitate was filtered and washed with diethylether (2 × 10 mL) and THF (2 × 10 mL), and then dried under vacuum to produce a white product. Yield: 0.44 g (73%) for **2**. Anal. Calcd. for C_13_H_13_Br_2_HgN_5_ (*M*_W_ = 599.69): C, 26.04; H, 2.19; N, 11.68%. Found: C, 26.32; H, 1.97; N, 12.01%. IR (KBr disk, cm^-1^): 3057(m), 2497(m), 1941(w), 1748(w), 1650(s), 1584(s), 1523(s), 1490(s), 1429(s), 1371(m), 1331(s), 1255(s), 1200(s), 1106(m), 1011(s), 981(m), 828(s), 594(m), 530(m). Yield: 0.49 g (71%) for **3**. Anal. Calcd. for C_13_H_13_HgI_2_N_5_ (*M*_W_ = 693.67): C, 22.51; H, 1.89; N, 10.10%. Found: C, 22.50; H, 2.03; N, 10.07%. IR (KBr disk, cm^-1^): 2936(w), 1669(m), 1587(s), 1570(m), 1491(m), 1474(w), 1391(w), 1314(m), 1222(w), 1202(m), 1057(w), 1010(m), 925(w), 828(m), 658(w), 614(w), 531(w). Crystals of **2** and **3** suitable for X-ray structural analysis were obtained by layering a MeCN solution of HgBr_2_ or HgI_2_, with a THF solution of 4-Hpyf.

### 2.4. X-ray Crystallography

The diffraction data for 4-Hpyf and complexes **1**–**3** were collected on a Bruker AXS SMART APEX II CCD diffractometer at 22 °C, which was equipped with a graphite-monochromated MoK_α_ (λ_α_ = 0.71073 Å) radiation. The structure factors were obtained after Lorentz and polarization. An empirical absorption correction based on “multi-scan” was applied to the data [16]. The positions of some of the heavier atoms, including the Hg atom, were located by the direct method or Patterson method of the SHELXS program, and the remaining atoms were found in a series of alternating difference Fourier maps and least-square refinements, while the hydrogen atoms were added by using the HADD command and refined as riding atoms [17]. The refined model for 4-Hpyf only represents one of several possible orientations. Basic information pertaining to crystal parameters and structure refinement is summarized in Table 1. Selected bond distances and angles are listed in Table 2.

### 2.5. Computational Methods

The density functional theory (DFT) calculations were performed for complexes **1**–**3** at the rb3lyp/lanl2dz level [18], which was implemented using the Gaussain 09 software package [19]. Coordinates of these complexes were obtained directly from X-ray crystallography.

### 2.6. Gas Adsorption Measurements

The adsorption isotherms for N_2_, H_2_, and CO_2_ were carried out on a Micrometrics ASAP 2020 Series analyzer by using gases of the highest quality at 77 and 293 K in a liquid nitrogen bath and an ice-water bath, respectively. Before the measurement, the sample was degassed (10^−3^ torr) at 423 K overnight to remove the co-crystallized solvent molecules.

## 3. Results and Discussion

### 3.1. Structure of 4-Hpyf·0.16THF

**Figure 2:**
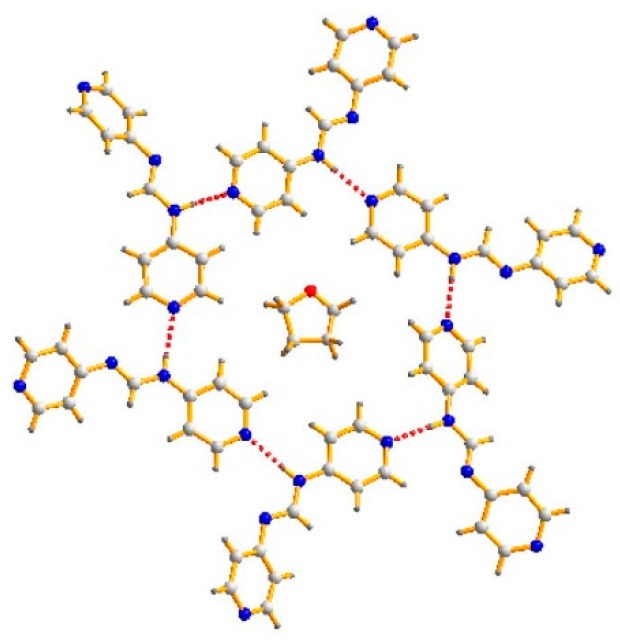
A packing diagram exhibiting the supramolecular structure of 4-Hpyf, showing that the molecules are linked through N–H···N hydrogen bonds.

The asymmetric unit of 4-Hpyf·0.16THF contains six 4-Hpyf molecules and one THF molecule. Figure 2 depicts a packing diagram showing that the six 4-Hpyf molecules are interlinked through N–H···N hydrogen bonds [H···N = 2.019–2.075 Å, ∠N–H···N = 168.4–179.2°] to form a ring, with the THF molecule occupying the center. It is noted that the structure of 4-Hpyf without co-crystallization of solvent has been reported, in which the 4-Hpyf molecules are linked by the similar N–H···N hydrogen bonds that results in a 1D linear chain [20]. The difference in the supramolecular structures indicates the structure-directing role of the THF solvent molecule.

### 3.2. Structure of ***1***

**Figure 3:**
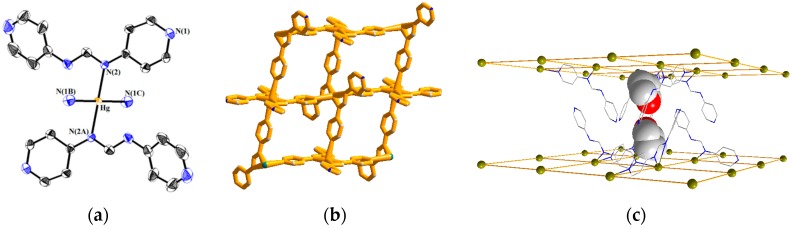
(**a**) Coordination environment about the Hg(II) ion of **1**; (**b**) A 2D layer structure for **1** showing the outward dangling nitrogen atoms. Thermal ellipsoids are shown at 30% probability level; (**c**) A packing diagram of **1** showing the intercalation of THF.

Figure 3a displays a drawing showing the coordination environment about the Hg(II) ion of **1**. The Hg(II) ion is coordinated by two amine nitrogen atoms [Hg–N = 2.073(4) Å] and two pyridyl nitrogen atoms [Hg–N = 2.691(6) Å] from four different 4-pyf^-^ ligands, resulting in a square planar geometry. The Hg–N distance of 2.691(6) Å is significantly shorter than the sum of the van der Waals radius of Hg and N atoms, which is 3.65 Å, indicating a strong interaction. Noticeably, the 4-pyf^-^ ligand coordinates to the Hg(II) ions through one amine and one adjacent pyridyl nitrogen atoms, resulting in a unique bidentate binding mode, Figure 1e. Subsequently, the Hg(II) ions are linked by a series of Hg–N bonds to form a 2D network, Figure 3b, which can be simplified to a 4-connected 2D net {4^4^·6^2^}-**sql** topology determined using TOPOS [21]. The area of each of the window is 8.26 × 8.26 Å^2^. Figure 3c shows that the THF solvent molecules are located in the space between the 2D layers, and the solvent-accessible volume calculated by PLATON program [22] is 605.7 Å^3^, which is 39.8 % of the unit cell volume.

### 3.3. Structures of ***2*** and ***3***

**Figure 4:**
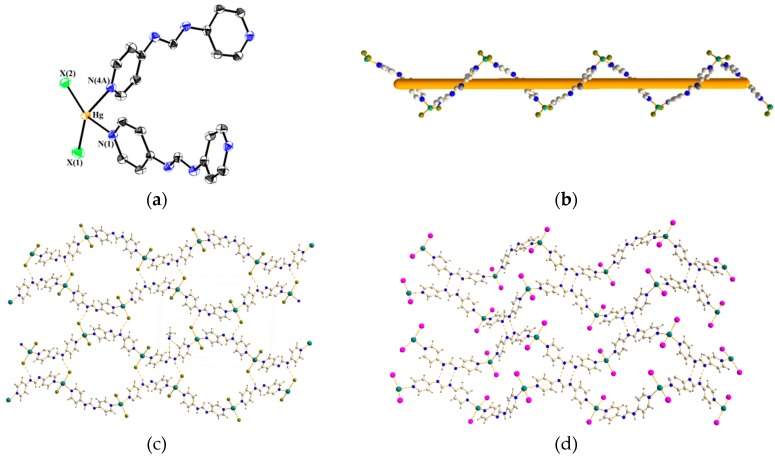
(**a**) A representative coordination environment about the Hg(II) ion for **2** (X = Br) and **3** (X = I); Thermal ellipsoids are shown at 30% probability level; (**b**) A representative drawing showing the 1D helical chain for **2** and **3**; (**c**) A packing diagram for **2**; (**d**) A packing diagram for **3**.

Figure 4a displays a representative drawing showing the coordination environment about the Hg(II) ion of **2** (X = Br) and **3** (X = I). Each of the Hg(II) ions is coordinated by two pyridyl nitrogen atoms [Hg–N = 2.358(5)–2.372(5) Å for **2** and 2.439(8)–2.451(7) Å for **3**] from two 4-Hpyf ligands and two halide anions, resulting in distorted tetrahedral geometries and forming 1D helical chains, Figure 4b. The neutral 4-Hpyf ligand adopts a new bidentate binding mode that coordinates to the Hg(II) ions through two pyridyl nitrogen atoms, Figure 1f. The halide anions show distinct effect on the supramolecular structures of **2** and **3**, Figure 4c,d. While the helical chains of **2** are linked by the N–H···Br (H···Br = 2.788 Å; ∠N–H···Br = 146.4°) hydrogen bonds, those of **3** are linked by the self-complementary N–H···N double hydrogen bonds (H···N = 2.186 Å; ∠N–H···N = 168.0°), resulting in 2D supramolecular structures for both complexes. The 2D supramolecular structures can be simplified as 4-connected 2D nets with the {4^4^·6^2^}-**sql** topology.

### 3.4. PXRD Patterns and Thermal Properties

Figures S2–S4 (Supplementary Material) show that the powder patterns of complexes **1**–**3** match quite well with those simulated from X-ray crystallography, indicating the bulk purities of these complexes.

The thermogravimetric analyses (TGA) were performed for (4-Hpyf)_6_·THF and **1** to examine their thermal stabilities, which were carried out in nitrogen atmosphere from 30 to 900 °C, Figure S5. The TGA curve of 4-Hpyf·1/6THF shows the gradual weight loss of THF of solvent molecules (calculated 5.7%; observed 5.5%) in 140–200 °C. The weight loss in 230–350 °C corresponds to the decomposition of 4-pyf^-^ ligand (calculated 94.3%; observed 94.1%). The TGA curve of **1** shows the gradual weight loss of THF of solvent molecules (calculated 19.5%; observed 19.1%) in 140–215 °C, and the weight loss of 53.6% in 270–420 °C corresponds to the decomposition of 4-pyf^-^ ligand (calculated 53.4%). The higher decomposition temperature range for the 4-pyf^-^ ligand in **1** than for the 4-Hpyf ligand in 4-Hpyf·1/6THF indicates the Hg–N bond is much stronger than the N–H···N hydrogen bond.

### 3.5. Luminescent Properties

**Figure 5:**
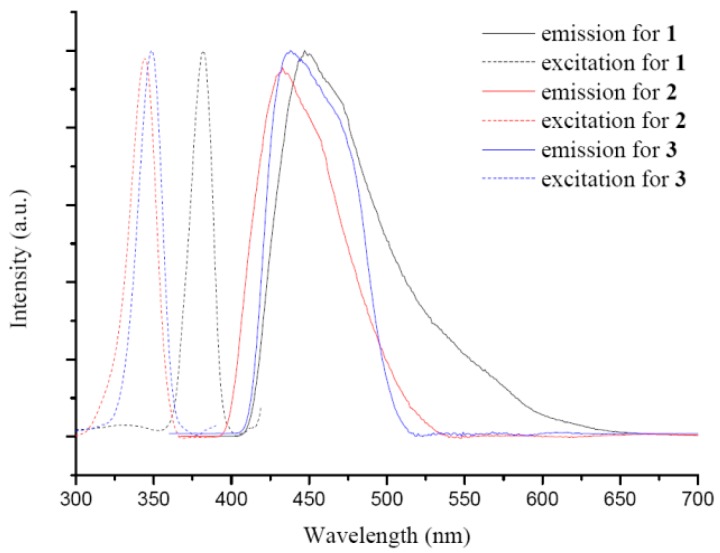
The emission and excitation spectra for complexes **1**–**3**.

Luminescent metal complexes are able to enhance, shift, and quench the luminescent emission of organic ligands through metal coordination. The emission and excitation spectra of **1**–**3** were measured in the solid-state at room temperature, Figure 5. The emission spectrum of **1** exhibits a broad band at 448 nm upon excitation at 381 nm, and those of **2** and **3** show emissions at 432 and 445 nm upon excitation at 344 and 348 nm, respectively. The difference in the emission wavelengths between **2** and **3** can be ascribed to the different electronegativity of the halide anions.

**Figure 6:**
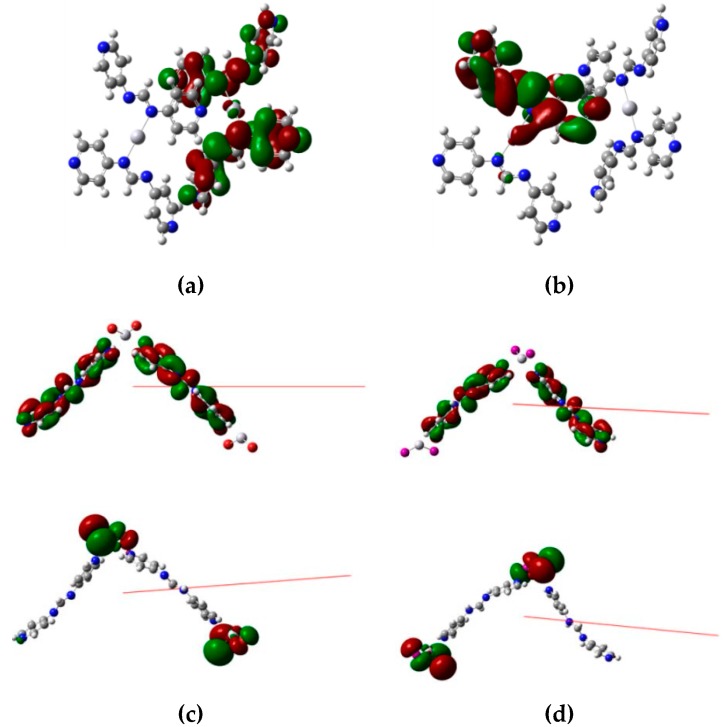
Drawings showing the electron contribution of (**a**) the highest occupied molecular orbital (HOMO) of **1**, (**b**) the lowest unoccupied molecular orbital (LUMO) of **1**, (**c**) HOMO (bottom) and LUMO (top) of **2**, and (**d**) HOMO (bottom) and LUMO (top) of **3**.

The results of the density functional theory (DFT) calculations show that the highest occupied molecular orbital (HOMO) and the lowest unoccupied molecular orbital (LUMO) energy gaps of **1**–**3** are 3.62, 3.84, and 3.80 eV, respectively, which match quite well with the excitation spectra. The results show that the HOMO and LUMO of **1** are derived from the π and π * orbitals of two different 4-pyf^-^ ligands, whereas those of **2** and **3** are primarily composed of non-bonding p-type orbitals from halide anions and the π * orbitals of the 4-pyf^-^ ligands, Figure 6 and Table 3. The emission of **1** is thus due to intraligand π→π * charge transfer between two different 4-pyf^-^ ligands, and those of **2** and **3** can be ascribed to the charge transfer from non-bonding p-type orbitals of the halide anions to π * orbitals of the 4-pyf^-^ ligands (*n*→π *).

### 3.6. Gas Sorption Studies

To investigate the effect of the dangling pyridyl nitrogen atoms on the pore structure and gas storage capability, gas sorption experiments were carried for the desolvated products of **1**, **1’**, and compared with the desolvated products of the Cu(I) coordination polymers *syn*-{[Cu_4_(4-pyf)_4_]·2EtOH}*_n_*, **4,***syn*-{[Cu_3_(4-pyf)_2_](BF_4_)·2H_2_O·EtOH}*_n_*, **5a** and *syn*-{[Cu_3_(4-pyf)_2_](ClO_4_)·EtOH}*_n_*, **5b**, *i.e.*, **4’**, **5a’** and **5b’**, respectively. The structures of the 2D layer **4** and the 3D frameworks **5** have been reported in a previous report, in which the 4-pyf ligands of **4** adopt the κ^3^-binding mode with one dangling pyridyl nitrogen atom, Figure 1d, while all the four nitrogen atoms of the 4-pyf ligands of **5** are coordinated to the metal centers, Figure 1c [15]. The solvent-accessible volume of **1**, **4**, **5a** and **5b** are 39.8%, 25.4%, 27.1%, and 33.3%, respectively, of their unit cell volume. Figures S2 and S6,S7 show that the powder patterns of **1’**, **5a’**, and **5b’** match well with the simulated patterns of **1**, **5a**, and **5b** obtained from single-crystal X-ray data, indicating no structural change during solvent removal. The pattern of **4’** can be regarded as a combination of the two phases of *syn*-[Cu_4_(4-pyf)_4_]*_n_*, Figures S8 [15].

**Figure 7:**
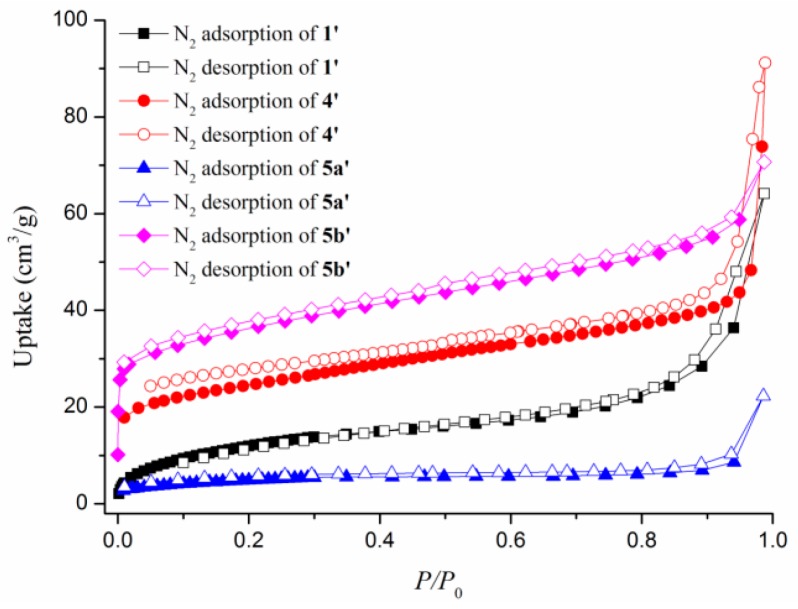
The N_2_ adsorption isotherms for **1’**, **4’**, **5a’**, and **5b’**.

**Figure 8:**
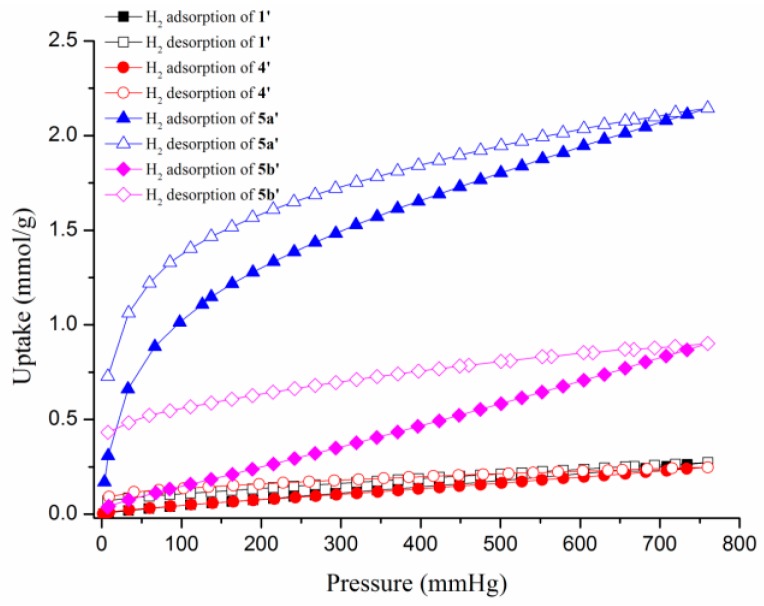
The H_2_ adsorption isotherms for **1’**, **4’**, **5a’**, and **5b’**.

**Figure 9:**
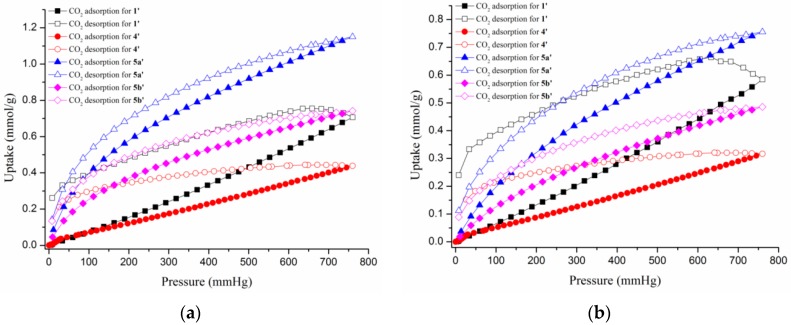
The CO_2_ adsorption isotherms for **1’**, **4’**, **5a’**, and **5b’** at (**a**) 273 and (**b**) 298 K.

The permanent macropore features are established by N_2_ adsorption isotherms at 77 K, which show the H3 type hysteresis loop for **1’** and **4’**, and typical type-II sorption behavior for **5a’** and **5b’** (Figure 7), and the maximum adsorption volumes were 64.14, 91.19, 22.31, and 70.68 cm^3^/g at *P*/*P*_0_ = 0.99, respectively. The Langmuir surface areas are determined by the linear fitting of N_2_ adsorption branch data and the result is 77, 154, 99, and 61 m^2^/g (BET surface areas are 45, 146, 94, and 59 m^2^/g) at 77 K. The amounts of hydrogen adsorption increase gradually with increasing hydrogen pressure and reveal maximum sorption amounts of 0.27, 0.25, 2.14, and 0.90 mmol/g for **1’**, **4’**, **5a’**, and **5b’** at 760 mmHg, respectively (Figure 8). The CO_2_ uptakes of **1’**, **4’**, **5a’**, and **5b’** at 273 and 760 mmHg are 0.71, 0.44, 1.15, and 0.74 mmol/g, respectively, which are lower than N_2_ adsorption (Figure 9a). The CO_2_ uptake capacity at 298 K is shown in Figure 9b. The different uptake amounts in the gas adsorption for **5a’** and **5b’** indicate that the nature of the BF_4_^-^ and ClO_4_^-^ anions are important in determining the adsorption capability.

**Figure 10:**
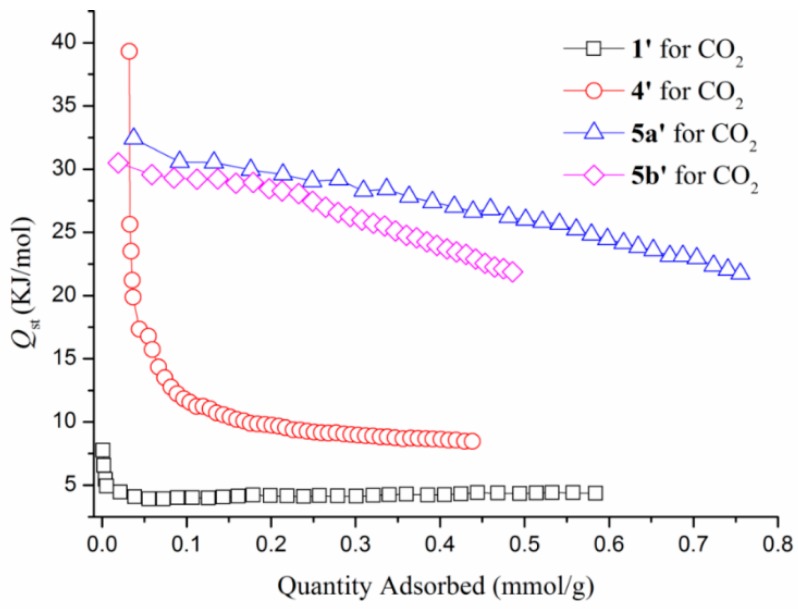
Isosteric heat of adsorption of **1’**, **4’**, **5a’**, and **5b’** at different CO_2_ uptakes.

The Clausius–Clapeyron equation implemented in the software of the Micrometrics ASAP 2020 sorptometer was employed to calculate the isosteric heats of CO_2_ adsorption (*Q*_st_) (Figure 10). The *Q*_st_ against loading amount was found to be 7.75 KJ/mol for **1’** at 0.0013 mmol/g, which displays the weak adsorption for CO_2_ between the layers of **1’**. 2D coordination polymers are often less stable with temperature and pressure variation than 3D ones during adsorption investigation because 2D coordination polymers usually present the supramolecular structure linked by hydrogen bonds. Presumably, the porosity of **1’** may have changed at higher gas pressure. The poorer data for **1’** may indicate the overloading of CO_2_ in the range of 600–760 mmHg, where saturation is reached. The *Q*_st_ of **4’**, **5a’**, and **5b’** exhibits maximum values of 39.31, 32.41, and 30.50 KJ/mol at near zero CO_2_ coverage and decreases with increasing CO_2_ loading amounts. Note that these are higher than the enthalpy of liquefaction of CO_2_ (17 kJ/mol) but lower than the values observed for zeolites such as NaX and Na-ZSM-5 at zero coverage (*ca.* 50 kJ/mol) [23,24,25], indicating relatively strong interactions between CO_2_ and the pore surfaces of **4’**, **5a’**, and **5b’**. The adsorption enthalpy leveled off rapidly when the loading amounts were over 0.039 mmol/g for **1’** and 0.051 mmol/g for **4’**. When the CO_2_ coverage increases, the *Q*_st_ remains steadily above 4.35 KJ/mol for **1’** and 8.46 KJ/mol for **4’**, respectively.

To compare the *Q*_st_ values for the 2D coordination networks of **1’** and **4’**, we propose that the outward dangling pyridyl nitrogen atoms may present the weak interactions with CO_2_ at zero coverage. From the *Q*_st_ values, it is found that **4’**, which has a smaller solvent-accessible volume, presents the larger *Q*_st_ value in CO_2_ adsorption. By DFT calculations, it can be shown that the Mulliken charges of the outward-dangling pyridyl nitrogen atoms (Figures S9 and S10, Supplementary Material) are 0.028 and 0.0033 for **1’** and -0.135 and -0.169 for **4’**, respectively. The smaller Mulliken charges of **1’** probably cannot invoke the interactions for any gas molecules and displays the physical adsorption for CO_2_. The larger CO_2_ uptake amounts for **1’** as compared with **4’** is thus probably due to the larger solvent-accessible volume with a more attractive surface to the CO_2_ molecules.

## 4. Conclusions

The reactions of 4-Hpyf with Hg(II) halide salts afforded three coordination polymers showing 2D net and 1D chains, in which the 4-pyf^−^ anions and the neutral 4-Hpyf ligands adopt the new bidentate binding modes. The formation of **1**–**3** indicates that the Cl^-^ anion is more readily removed by the amine hydrogen atom of the 4-Hpyf ligand than the Br^-^ and I^-^ anions. The halide anions also show significant effect on the supramolecular structures of **2** and **3**. While the helical chains of **2** are linked through N–H···Br hydrogen bonds, those of **3** are linked through self-complementary double N–H···N hydrogen bonds. Structural comparisons of **1**–**3** show that deliberate choice of the starting Hg(II) halide salts is important in controlling the folding and unfolding of the Hg(II) coordination polymers in terms of topology. Density function theory (DFT) calculation indicates that the emission of **1** is due to intraligand π→π * charge transfer, and those of **2** and **3** are due to the *n*→π * charge transfer. It is also shown that the nature of the BF_4_^−^ and ClO_4_^−^ anions in **5a** and **5b** and the larger solvent-accessible volume of **1** are important in determining the adsorption capability. However, the effect of the electron densities on the dangling nitrogen atoms cannot be overlooked.

## Figures and Tables

**Table 1:** Crystallographic data for 4-Hpyf·0.16THF and **1**–**3**.

	4-Hpyf·0.16THF	1	2	3
Formula	C_11.67_H_11.33_N_4_O_0.17_	C_30_H_34_HgN_8_O_2_	C_13_H_13_Br_2_HgN_5_	C_13_H_13_HgI_2_N_5_
fw	210.25	739.24	599.69	693.67
Space group	*P*ī	*P*2_1_/*c*	*P*2_1_/*c*	*P*2_1_/*c*
*a*, Å	10.7902(1)	11.3357(3)	4.9204(1)	5.1343(2)
*b*, Å	16.2328(2)	12.7290(3)	21.3903(4)	21.1146(9)
*c*, Å	19.7864(2)	10.5421(2)	16.2077(3)	16.6380(8)
α, deg	101.218(1)	90	90	90
β, deg	93.271(1)	91.331(1)	92.653(1)	96.902(2)
γ, deg	106.720(1)	90	90	90
*V*, Å3	3,232.10(6)	1,520.73(6)	1,704.01(6)	1,790.63(13)
*Z*	12	2	4	4
*D*_calc_, g·cm^−3^	1.296	1.614	2.338	2.573
μ, mm^−1^	0.084	5.101	13.722	12.044
No. of reflns meased	51,996	13,961	17,060	13,570
Independent reflections *R*_int_	12,722 (0.0331)	2,977 (0.0400)	3,321 (0.0518)	3,526 (0.0650)
No. of params	856	182	194	190
Quality-of-fit indicator ^c^	1.040	1.087	1.053	1.069
Final *R* indices [*I* > 2σ(I)] ^a,b^	*R*_1_ = 0.0578, *wR*_2_ = 0.1478	*R*_1_ = 0.0537, *wR*_2_ = 0.1788	*R*_1_ = 0.0363, *wR*_2_ = 0.1014	*R*_1_ = 0.0504, *wR*_2_ = 0.1246
*R* indices	*R*_1_ = 0.1142	*R*_1_ = 0.0651	*R*_1_ = 0.0482	*R*_1_ = 0.0634
(All data)	*wR*_2_ = 0.1789	*wR*_2_ = 0.1971	*wR*_2_ = 0.1077	*wR*_2_ = 0.1313

^a^ *R*_1_ = Σ||*F*_o_| − |*F_c_*||/Σ|*F*_o_|. ^b^ *wR*_2_ = [Σ *w*(*F*_o_^2^ – *F_c_*^2^)^2^/Σ *w*(*F*_o_^2^)^2^]^1/2^. *w* = 1/[σ^2^(*F*_o_^2^) + (ap)^2^ + (bp)], *p* = [max(*F*_o_^2^ or 0) + 2(*F_c_*^2^)]/3. *a* = 0.0877, *b* = 0.4074 for 4-Hpyf·0.16THF; *a* = 0.1426, *b* = 0.0000 for **1**; *a* = 0.0661, *b* = 0.0000 for **2**; *a* = 0.0740, *b* = 0.0000 for **3**. ^c^ quality-of-fit = [Σ *w*(|*F*_o_^2^| − |*F_c_*^2^|)^2^/*N*_observed_ − *N*_parameters_ )]^1/2^.

**Table 2:** Selected bond lengths (Å) and angles (deg) for **1**, **2** and **3**.

**1**			
Hg–N(2)	2.075(5)	N(2)–Hg–N(2A)	180.0(2)
Hg–N(2A)	2.075(5)	N(2)–Hg–N(1B)	94.3(2)
Hg–N(1B)	2.690(6)	N(2A)–Hg–N(1B)	85.7(2)
Hg–N(1C)	2.690(6)	N(2)–Hg–N(1C)	85.7(2)
		N(2A)–Hg–N(1C)	94.3(2)
		N(1B)–Hg–N(1C)	180.0
**2**			
Hg–N(4A)	2.362(5)	N(4A)–Hg–N(1)	108.04(19)
Hg–N(1)	2.366(5)	N(4A)–Hg–Br(1)	102.77(14)
Hg–Br(1)	2.4923(8)	N(4A)–Hg–Br(2)	99.27(13)
Hg–Br(2)	2.5425(8)	N(1)–Hg–Br(1)	100.26(13)
		N(1)–Hg–Br(2)	97.16(13)
		Br(1)–Hg–Br(2)	145.94(3)
**3**			
Hg–N(4A)	2.450(7)	N(4A)–Hg–N(1)	105.6(3)
Hg–N(1)	2.439(8)	N(4A)–Hg–I(1)	97.9(2)
Hg–I(1)	2.6541(8)	N(4A)–Hg–I(2)	102.0(2)
Hg–I(2)	2.6415(8)	N(1)–Hg–I(1)	99.58(18)
		N(1)–Hg–I(2)	98.41(18)
		I(1)–Hg–I(2)	148.40(3)

A: −x + 2, −y + 1, −z; B: −x + 2, y − 1/2, −z + 1/2; C: x, −y + 3/2, z − 1/2 for **1**. A: −x, y + 1/2, −z + 3/2 for **2**. A: −x + 2, y − 1/2, −z + 3/2 for **3**.

**Table 3:** The energies (eV) for HOMO and LUMO of complexes **1**–**3**.

	1	2	3
LUMO+2	−1.92	−1.85	−1.85
LUMO+1	−2.28	−2.79	−2.61
LUMO	−2.54	−2.82	−2.64
HOMO	−6.17	−6.66	−6.44
HOMO−1	−6.26	−6.66	−6.44
HOMO−2	−6.63	−6.68	−6.45

## Supplementary Materials

Supplementary materials can be found at www.mdpi.com/2073-4360/8/4/137/s1. Crystallographic data for 4-Hpyf and **1–3** have been deposited with the Cambridge Crystallographic Data Centre, CCDC No. 1441868-1441871.
