# Supplementary material for: Hg(II) Coordination Polymers Based on N,N’-bis(pyridine-4-yl)formamidine
Source: Polymers (Basel). 2016 Apr 11;8(4):137. doi: 10.3390/polym8040137 (PMC6432418; doi:10.3390/polym8040137)
Supplement: Supplementary file 1 [file polymers-08-00137-s001.pdf]

Supplementary Materials: Hg(II) Coordination Polymers Based on *N,N′*-bis(pyridine-4-yl)formamidine

Wayne Hsu, Xiang-Kai Yang, Pradhumna Mahat Chhetri and Jhy-Der Chen

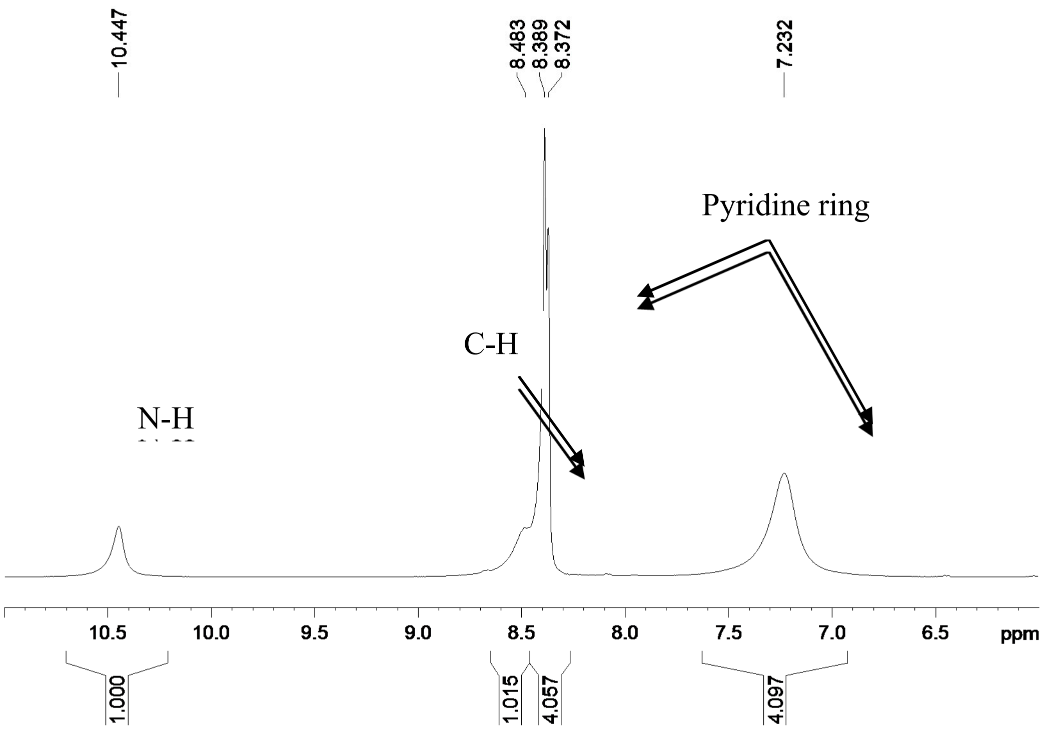

Figure S1. The ^{1}H NMR spectrum of 4-Hpyf.

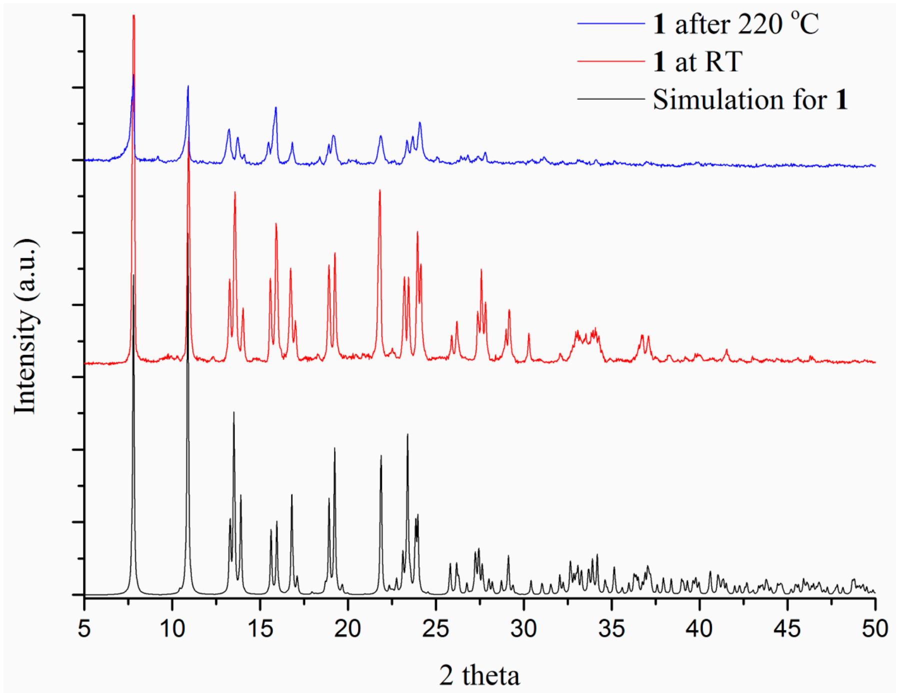

Figure S2. The PXRD patterns for complex **1**.

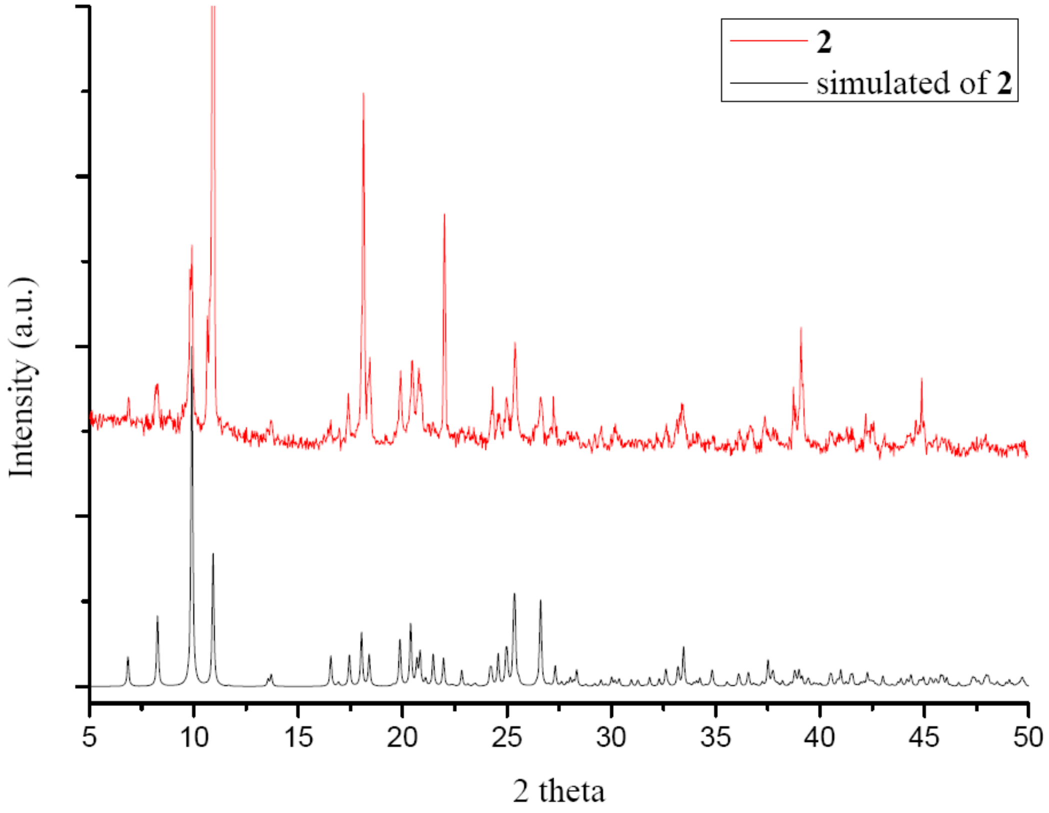

Figure S3. The PXRD patterns for complex **2**.

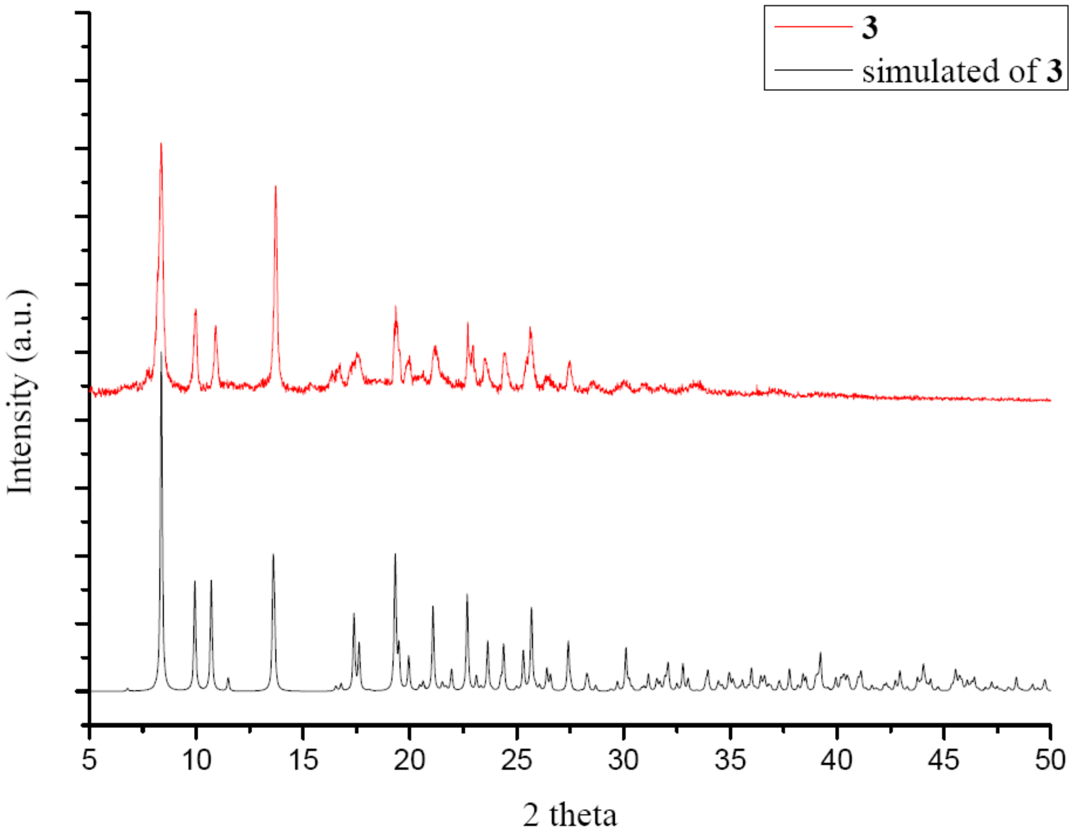

Figure S4. The PXRD patterns for complex **3**.

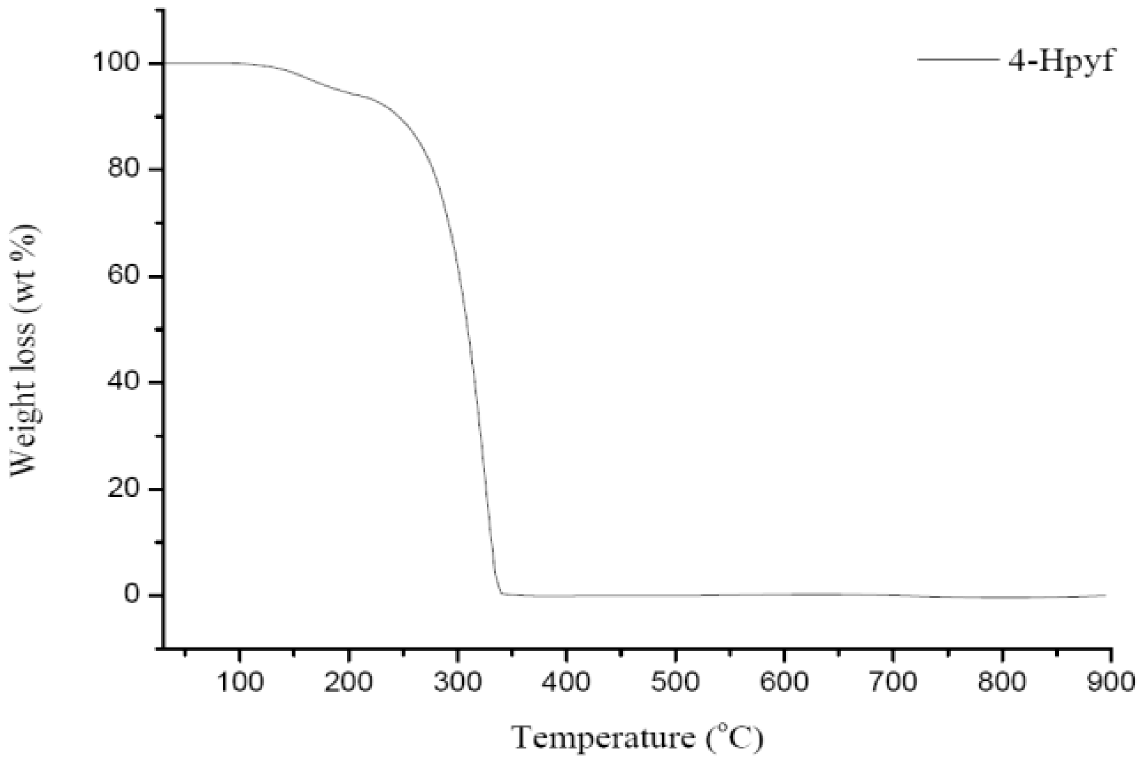

(**a**)

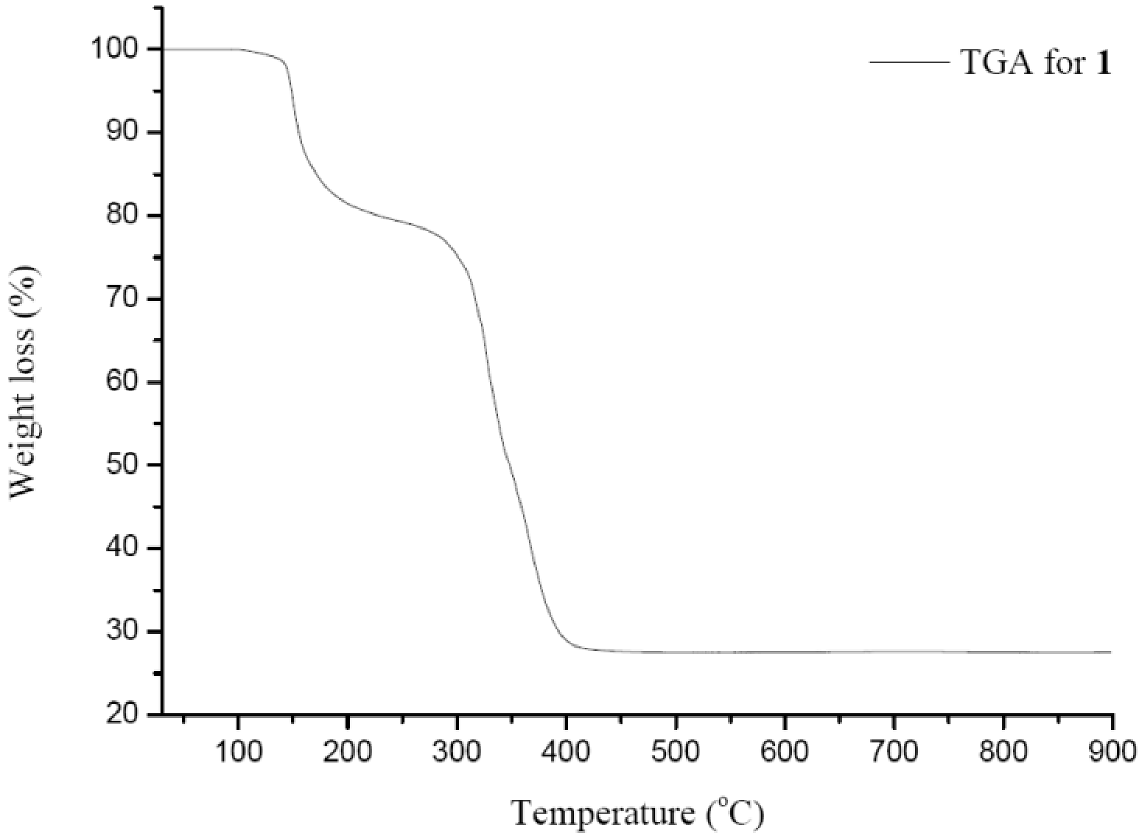

(**b**)

Figure S5. TGA curves for (**a**) 4-Hpyf and (**b**) **1**.

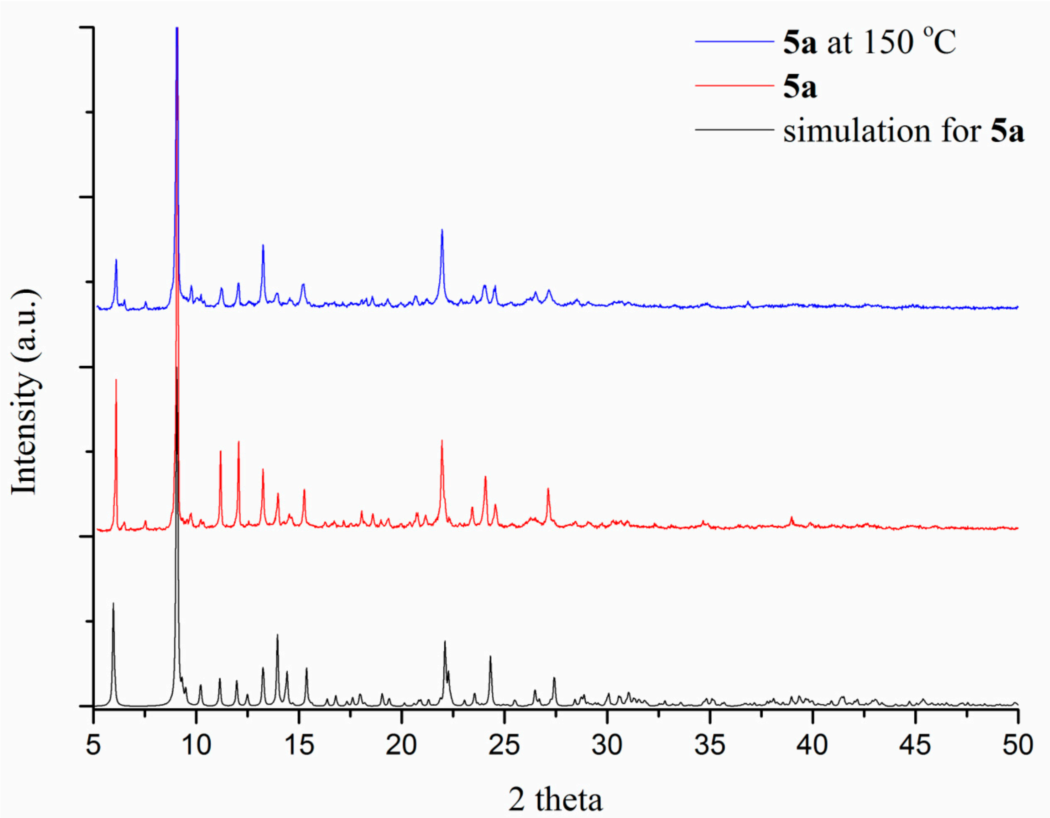

Figure S6. Simulated, experimental and 150 °C powder XRD patterns for **5a**.

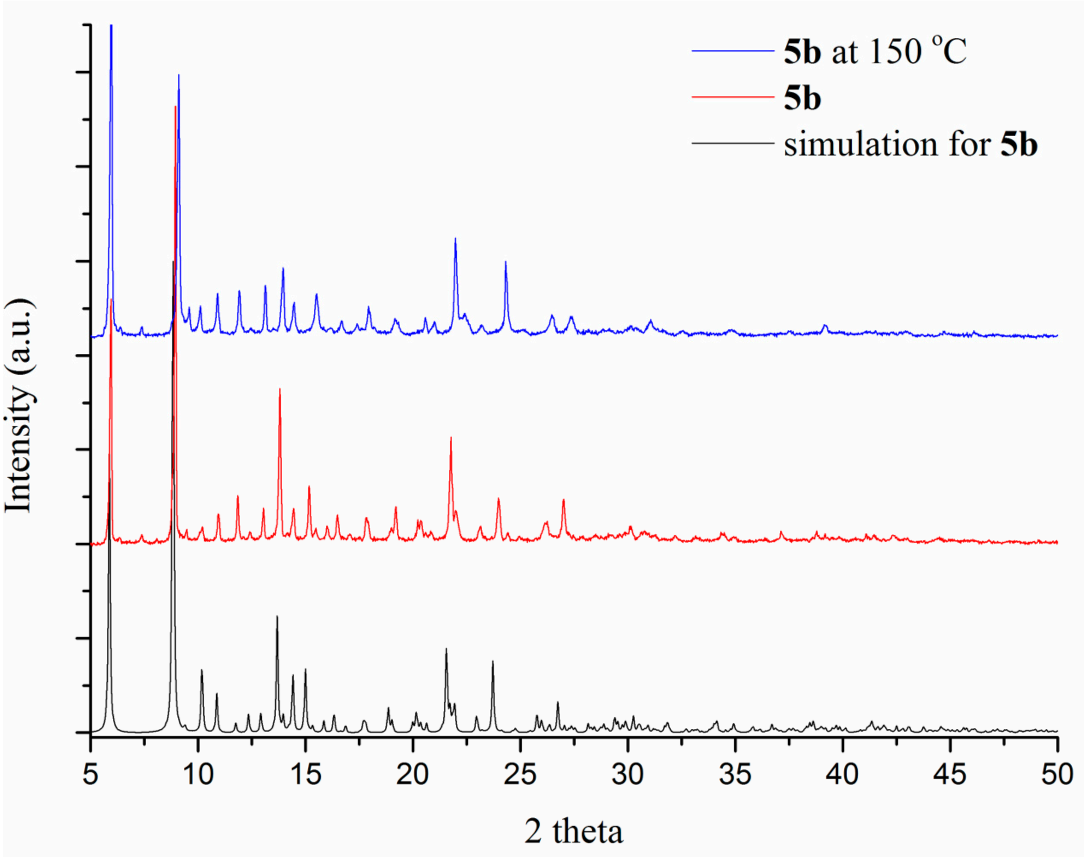

Figure S7. Simulated, experimental and 150 °C powder XRD patterns for **5b**.

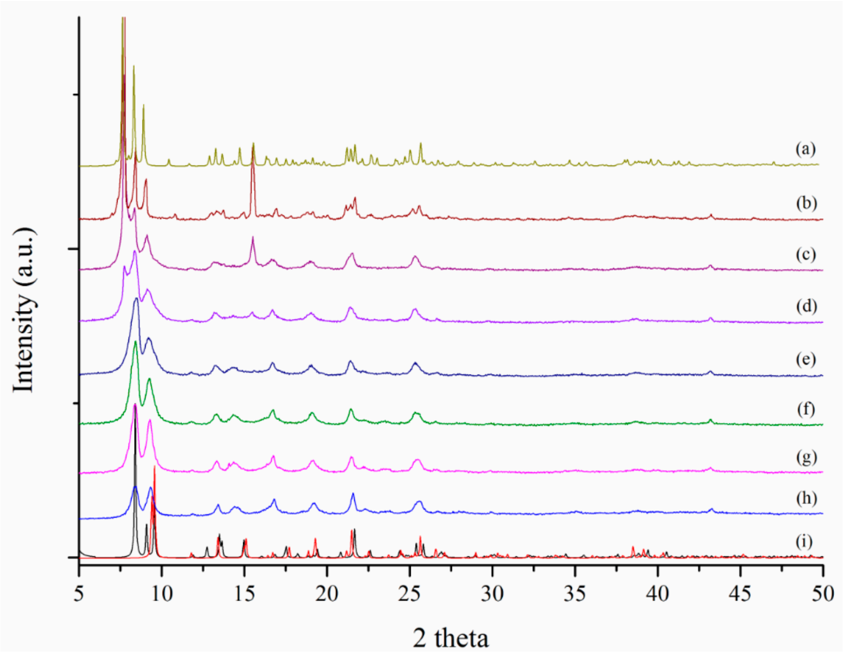

Figure S8. (**a**) Simulated PXRD patterns for 4 and patterns at (**b**) 30 °C, (**c**) 90 °C, (**d**) 100 °C, (**e**) 120 °C, (**f**) 160 °C, (**g**) 200 °C, (**h**) 240 °C. (**i**) Combined simulated patterns for *syn*-[Cu(4-pyf)]n.

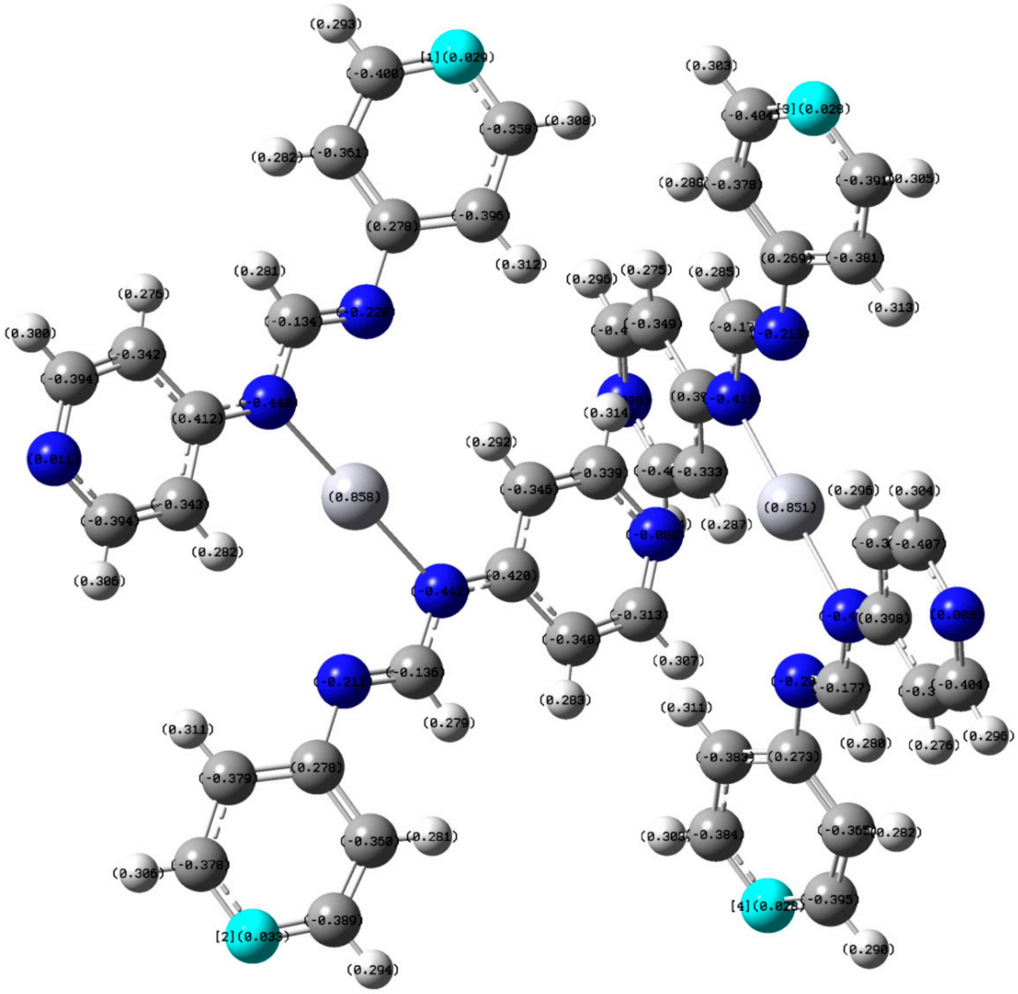

Figure S9. The Mulliken charge in asymmetric units for complex **1**. The outward dangling pyridyl atoms are shown with light blue color.

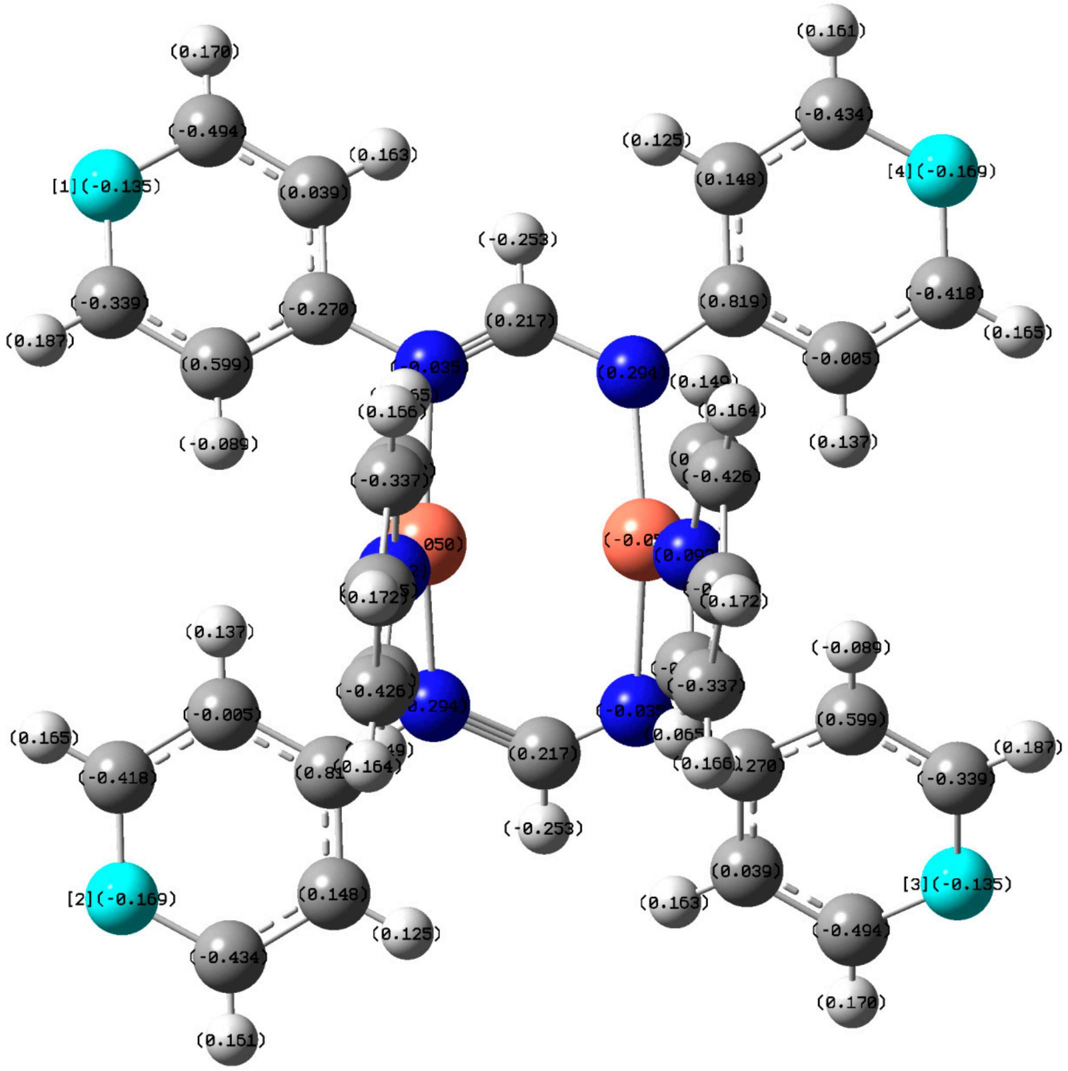

Figure S10. The Mulliken charge in asymmetric units for complex **4**. The outward dangling pyridyl atoms are shown with light blue color.

© 2016 by the authors; licensee MDPI, Basel, Switzerland. This article is an open access article distributed under the terms and conditions of the Creative Commons by Attribution (CC-BY) license (http://creativecommons.org/licenses/by/4.0/).
